# Teachers’ experiences with the Back2School intervention—a pilot study addressing problematic school absenteeism

**DOI:** 10.3389/fpsyg.2025.1608184

**Published:** 2025-08-04

**Authors:** Elisabeth Valmyr Bania, Toril Sørheim Nilsen, Mikael Thastum, Jo Magne Ingul, Trude Havik

**Affiliations:** ^1^The Regional Centre for Child and Youth Mental Health and Child Welfare – Central Norway, RKBU Midt-Norge, Norwegian University of Science and Technology (NTNU), Trondheim, Norway; ^2^Institute of Psychology, The Arctic University of Norway, Tromsø, Norway; ^3^Department of Psychology and Behavioral Sciences, Aarhus University, Aarhus, Denmark; ^4^Norwegian Centre for Learning Environment and Behavioural Research, University of Stavanger, Stavanger, Norway

**Keywords:** school absenteeism, CBT intervention, Back2School, teacher involvement, teacher experiences

## Abstract

**Introduction:**

School absenteeism represents a concern for students, educators, and parents alike. Teachers’ involvement is vital to students’ school life. Consequently, integrating schools and teachers effectively in absenteeism interventions is of great importance. However, few studies have investigated teachers’ perspectives on participating in manual-based, indicated interventions to promote school attendance. This study aimed to explore teachers’ experiences with the manual-based Back2School (B2S) intervention, which is based on cognitive behavioural therapy (CBT).

**Methods:**

Seven primary and lower secondary school teachers agreed to participate in individual interviews following their involvement in the intervention. These teachers engaged in various aspects of the intervention, including data collection, school sessions, and school meetings involving students, parents, and B2S group leaders.

**Results:**

The results indicate that some of the informants experienced increased competence and self-efficacy regarding school absenteeism following the intervention, while other informants did not have this experience.

**Discussion:**

There is a need for more clarity and enhanced teacher involvement in future B2S interventions.

## Introduction

Regular school attendance is a critical measure of educational success for young people (Gottfried et al., 2024). Despite this, school absenteeism constitutes a vexing problem in many countries (Chu et al., 2019; Fredriksson et al., 2023). Norway lacks a comprehensive national statistic on school absenteeism. However, the Directorate for Education has provided an overview indicating that 15 percent of Norwegian 10th graders (age 16) accumulated 10 percent or more days of absence during their last school year (UDir, 2023).

The field of school absenteeism is characterized by its complexity, featuring diverse terms, definitions of key concepts, functional conceptualizations, different cut-off values for when absenteeism is considered a problem requiring intervention, and a myriad of interpretive perspectives on understanding this topic (Havik and Ingul, 2021). Nevertheless, a consensus exists within the field regarding the importance of school attendance for students’ academic and social development, the multifaceted nature of the problem, and the necessity for improved interventions targeting this heterogeneous group of students (Kearney, 2021). School absenteeism also represents a public health concern (Fernandes et al., 2024), as it functions as a predictor of school dropout, later marginalization, and reduced quality of life (Archambault et al., 2022; Kearney and Graczyk, 2020).

Teachers play a pivotal role for students in all aspects of school life, including fostering a safe learning environment, influencing student engagement, learning, and well-being, and promoting school attendance (Graczyk and Kearney, 2024). However, determining appropriate actions when a student struggles with problematic school absenteeism poses significant challenges for many teachers and other school personnel (Kearney et al., 2023). Teachers often perceive addressing school absenteeism as emotionally demanding and resource-intensive, reporting that they dedicate disproportionate time to these students compared to the rest of the class (Finning et al., 2018; Kearney et al., 2022). Moreover, teachers express difficulties understanding absenteeism (Gren-Landell et al., 2015; Kearney et al., 2022), whereas parents may feel that schools lack competence and understanding regarding how to intervene and calls for a better-coordinated approach (Akkus and Çinkir, 2022; Havik et al., 2014). Other findings indicate that many teachers experience challenges regarding school absenteeism as it adds to their workload and negatively impact their morale when trying to help students catch up (Kearney et al., 2023; Malcolm et al., 2003; Wilson et al., 2008), which results in frustration and limited return on their efforts.

The present situation calls for improved involvement of schools and teachers in interventions addressing school absenteeism.

However, a meta-analytic review by Gubbels et al. (2019) identified potential risk factors for school absenteeism, where the following had large effects: negative attitude towards school, substance abuse, externalizing and internalizing problems, and low parent-school involvement. The three school factors found to have a significant effect on school absenteeism were poor pupil-teacher relationships, low quality of school/education, and a negative school/class climate, operationalized as both individual risk factors and domains of risk factors (Gubbels et al., 2019; Lomholt et al., 2022). These numerous potential risk factors indicate the importance of adopting a holistic view to understand and intervene, where all stakeholders’ roles must be considered and integrated in interventions (Graczyk and Kearney, 2024; Kearney et al., 2023; Melvin et al., 2025).

Several systematic reviews and/or meta-analyses addressing interventions for school absenteeism mainly focus on CBT interventions. One systematic review included 78 interventions designed for school refusal or truancy among children and youth aged 6–18 years old (Pérez-Marco et al., 2024). They found that CBT was the most frequently used method. In general, the effects of CBT interventions were larger among the youngest children than for adolescents. Research indicates that between one-third and two-thirds of young people with absenteeism do not respond to interventions (Heyne, 2022), this could be explained by adolescent developmental challenges such as increased academic and social demands, and higher rates of social anxiety and depressive disorders reaching adolescence. This highlights the need for more attention to students’ developmental sensitivity to improve interventions among adolescents (Heyne and Brouwer-Borghuis, 2022).

Weeks et al. (2017) investigated factors in the school setting as outcomes of a group CBT-based intervention in the UK. Student identification, measures of change applied, and the role of school staff was identified as influential factors. This implies the need to support school staff in identifying students who fit the intervention and allocate sufficient time for them to develop their knowledge and understanding of the intervention’s key aspects to promote students’ mental health and well-being (Chu et al., 2019; Graczyk and Kearney, 2024). The importance of close integration and cooperation with the family and school staff for a successful CBT intervention was emphasized several years ago (Elliott and Place, 2019; Heyne et al., 2015). However, based on previous reviews, there is still a need for more research on integrated CBT approaches. A systematic review by Boaler and Bond (2023) concluded that systemic approaches facilitated by schools were characterized by proactive systems, a supportive school ethos, personalized intervention, and collaboration with families. Schools exhibiting these characteristics were associated with promising outcomes related to increased attendance and engagement (Boaler and Bond, 2023). Findings from a recent systematic review by Fernandes et al. (2024) highlight key elements of absenteeism interventions, such as implementing CBT in school environments and incorporating a school-based component as improving effectiveness. They recommended further exploration and development of interventions with an integral school-based component.

Heyne and Brouwer-Borghuis (2022) identified 14 signposts for effective school refusal interventions, highlighting the essential role of schools and collaboration, and advocating for an integrated approach involving the student, parents, and school personnel to ensure coherence between home and school interventions. They also emphasize the need for structured, ongoing collaboration between schools and external support services. The contextual and relational nature of absenteeism, and the importance of viewing it as a network problem, were highlighted in a qualitative study by Nielsen and Thastum (2023), along with the significance of collaboration for resolving absenteeism. For teachers, the need for more knowledge and overcoming barriers in their everyday workload was identified as necessary to provide good support and collaboration with parents (Hejl et al., 2024; Nielsen and Thastum, 2023). Teachers emphasized the need for joint, interdisciplinary collaboration with external professionals and called for more structured support and guidance in managing individual cases of absenteeism problems (Hejl et al., 2024; Nielsen and Thastum, 2023). Moreover, teachers also reported that involving a third-party mediator could improve the management of absenteeism. The importance and need for a holistic approach, involving schools are clear. However, to the best of our knowledge, no previous studies have focused on teachers’ in-depth experiences when participating in a modular CBT intervention for students with problematic school absenteeism, except for the feasibility study of “Back2School” (B2S) (Lomholt et al., 2020). Students and parents were not interviewed in the present study, because an examination of this topic is the focus of a forthcoming study.

### The Back2School (B2S) intervention

The Back2School intervention was developed in Denmark (Johnsen et al., 2024; Thastum et al., 2020; Thastum et al., 2019) to address problematic school absenteeism, defined as more than 10 percent absenteeism over the preceding 3 months (Kearney and Childs, 2023). This cut-off aligns with other recommendations (Balfanz and Byrnes, 2012; Chang et al., 2018; Department for Education, 2024; Kirksey, 2019). Findings from the feasibility study of B2S in Denmark indicated that teachers were less satisfied than parents and youths, but the majority found the school meetings useful (Lomholt et al., 2020). Teachers suggested improvements such as involving the school earlier and clearer communication between the involved parties. The study highlighted the need for greater engagement and information, for teachers to improve their satisfaction and involvement in the B2S intervention (Lomholt et al., 2020). Both parents and teachers mentioned school involvement as an important part of B2S, but they wanted the school meetings scheduled earlier in the intervention (Lomholt et al., 2020). Other relevant findings from the feasibility study included the importance of involving school management, gathering more information about the student’s school class and social environment, and the need for clearer communication during the program. In the effectiveness study of B2S in Denmark, the intervention was compared to “treatment as usual” (TAU). Both B2S and TAU improved students’ school attendance, but B2S significantly outperformed TAU in reducing emotional, behavioral, and social problems in students, and increased school-related self-efficacy for both students and parents (Johnsen et al., 2024).

### The Norwegian B2S pilot study

Based on the findings from the Danish feasibility study (Lomholt et al., 2020), which was published before the intervention in the Norwegian pilot study commenced, the manual was expanded to involve and engage the school at an earlier stage and by adding two compulsory and one optional third school session. Additionally, the school was asked to complete questionnaires about the pupil’s attendance and mental health [Strengths and Difficulties Questionnaire (SDQ)] before, during, and after the intervention, as well as 2 weeks before the booster session. In total, the teachers registered students’ attendance rates for 6 months. The 13-week intervention consisted of 4 school meetings (with parents) and 2–3 school sessions (without parents). The school sessions were held between the B2S team and the school (the main teacher and someone from the school management), without the parents or student present, whereas the school meetings included all parties involved in the intervention (timeline available on request). The B2S pilot intervention was conducted with 14 students across Norway in 2022–2023. A multi-professional research group supervised members of the B2S team in eight municipalities in an individual indicative preventive intervention (The B2S intervention, manual available on request) for students in grades 4–10 with school absenteeism problems. The intervention took place at home, in school, or during leisure time. The B2S team members were employed by the municipality and had pedagogical, health, and/or family-related backgrounds, where several were dedicated to school absenteeism cases and worked in school absenteeism/attendance teams within the municipality. The aim of this study is to gain in-depth insight into the process behind teachers’ participation in piloting the Back2School intervention by investigating the following research question: How do teachers experience their participation in the Back2School (B2S) pilot intervention?

## Materials and methods

### Study design

The current study employed a qualitative design, conducted among teachers who participated in the pilot study of B2S. Based on the research question, a semi-structured interview guide was developed (available on request). The interviews were conducted by two researchers during spring 2023. All interviews were conducted digitally and audio-recorded securely, with the transcript data material stored at SAFE (Secured Access to Research Data and e-infrastructure), University of Bergen, Norway. Transcription was outsourced to a third party.

### Recruitment and sample

The starting point for recruitment was a list of the 14 teachers and Head of Department who had participated in a teacher role in the B2S pilot study. Seven of them consented to be contacted personally via phone and email with information and a request to participate in an individual qualitative interview (approximately 40 min). All seven responded positively. Six out of seven were main teachers for the participating student, and one was a Head of Department who participated in all the meetings and served as a teacher for the student. Initially, a pilot interview was conducted with an experienced psychologist from the B2S research group who was not involved in interviewing teachers. The aim was to get feedback on the time required and assess whether the questions were easy to follow and understand.

### Analysis

Based on the research question and the study’s exploratory-descriptive design (Bernacki et al., 2021; Hunter et al., 2019), thematic analysis was deemed a suitable method to code and analyse data (Braun and Clarke, 2006; Clarke and Braun, 2021). The analysis followed five steps: (1) data familiarization, (2) generating initial codes, (3) searching for themes, (4) reviewing themes, and (5) defining and naming themes. Two researchers coded the material separately. The codes were then reviewed and revised by adding, removing, and merging codes, and sorting them into different themes. The two researchers discussed and reached a joint agreement throughout the coding process. In the final phase, one of the researchers reviewed all the coding again and made further revisions to the codes and themes, before creating a matrix consisting of three main thematic categories and eight subcategories (see Table 1). An abductive approach was used in the current study (Thompson, 2022), combining a theory-driven, deductive approach with a more empirical, inductive approach (Clarke and Braun, 2021).

**Table 1:** The informants’ experiences: main themes and subthemes.

Main themes	Subthemes
School role and involvement in B2S	Entrance and recruitment
	Collaboration and inclusion of the school’s perspective
	Workload
	School sessions and school meetings
Impact of B2S participation	Teachers’ competence and self-efficacy
	Students’ attendance
Feedback on B2S	Success factors
	Barriers

### Trustworthiness

The results of this study are considered verifiable and reliable (Cohen et al., 2002; Nazar et al., 2023). Although the study concerns a specific Norwegian context, the findings may contribute valuable insights for similar interventions in comparable settings (Malterud, 2002; Stalmeijer et al., 2024). To ensure trustworthiness, two researchers performed the analysis independently at first, and then collaboratively. The authenticity of the study refers to the overall process, where all parts are transparent, thus enhancing transferability (Nazar et al., 2023). Further, to ensure authenticity, we prioritized transparency in sharing the details of our methods and analytical process.

### Ethical considerations

The B2S study has been approved by the Regional Committees for Medical and Health Research Ethics (REK) (ref. nr. 462,879). Written consent was obtained from both parents for the children’s participation. There is also a data processing agreement, and the data material is stored in accordance with regulations at the Services for Sensitive Data (SAFE), University of Bergen. This also includes teacher data, where the teachers provided consent to be interviewed after the completion of the pilot study. As teachers refer directly to individual students or third parties, there is an ethical risk associated with the material requiring careful anonymization during analysis and reporting.

## Results

The main findings are presented in Table 1 and organized into three main themes and eight subthemes. The informants are referred to as P1, P2, etc., based on the sample description in Table 2. Each subtheme is presented continuously, underpinned by informant quotes.

**Table 2:** Characteristics of the informants.

Informant	Years of experience	Student’s grade level	Role
P1	8	10th Grade	Main teacher
P2	7	9th Grade	Main teacher
P3	32	9th Grade	Main teacher/Head of School Department
P4	20	9th Grade	Main teacher
P5	18	8th Grade	Head of School Department
P6	3.5	6th Grade	Main teacher
P7	12	6th Grade	Main teacher

### School role and involvement in B2S

#### Entrance and recruitment

Two informants (P2, P5) experienced a messy entrance into the project. Informant P2 said it was back-and-forth in the beginning, as they were told they could participate, then they were told they could not, and finally, they were given the opportunity to participate. Informant P5 expressed that the school should be involved in the selection of which student should be included. Further, the same informant believed they had established a good intervention for the student at school and good communication with the parents before the B2S intervention, but asserted that this communication deteriorated during the B2S intervention:

“The student started and participated as usual and performed well. In that meeting, we were informed that it was not necessary to implement any attendance plan again because it was working so well. And the week after, B2S was introduced to this student. So, for us, it was a very strange approach to it.” (P5).

The other five informants did not mention difficulties with the entrance to B2S; however, they indicated many interventions had been attempted before the B2S intervention. When asked who the decision makers were for participation, two informants expressed that they were not certain or did not remember (P5, P1), illustrated by one of them:

“I do not really remember how it started, but we had been working on it for a while. There were many parties involved. I do not know who tipped them off about this student, I do not quite remember.” (P1).

#### Collaboration and inclusion of the school’s perspective

The importance of collaboration among all involved parties was emphasized by all the informants. They all expressed the importance of good school-home cooperation. Moreover, they valued being included with a team of external professionals (B2S team) for discussion and acknowledgment of their prior efforts. Most informants were pleased to be part of the B2S intervention, although they found it challenging to manage the required time commitment without additional resources. One quote illustrates the relief of not being solely responsible for the student’s situation:

“…helpless when dealing with such a difficult case. You can send a message to child welfare services, and it will be addressed there, but they often check the case after a few months. Then you have to send a new request if you need help from them, even if the parents were involved in the application. And then we tried to get in touch with BUP (Child and Adolescent Mental health outpatient Clinic), which is very difficult. So, getting external help means a lot. For the student and the other students, it helps to have someone with expertise in this area, but as a teacher, it was reassuring to know that you did not have to handle it alone, that you got help.” (P1).

Another informant highlighted the benefits for communication:

“Yes, it was very useful because we gained more insight into the measures being taken at home, and the parents became very confident in what we are doing at school. We also got to hear what the student says at home, and they got insight into what the student says at school. It goes back to that common understanding, clearing up communication, which has been good. And if we have disagreed or felt stuck or uncertain, we have a third party there who can help us get started.” (P6).

The informants noted the value of consulting with professionals with competence and experience in this topic. They said it meant a lot to get external help and felt supported in how to assist the student. Achieving a “common understanding” of the student’s situation and problem emerged as a key element in the intervention, where all parties involved worked together to understand the present situation, including risk and protective factors. The tool “step-ladder,” based on SMART goals as known from CBT treatment (Josefowitz and Myran, 2021), was mentioned by several informants as a useful concept.

“At least some tools were highlighted, like the ‘step-ladder’. There are many great and exciting ideas. Some of the tools, if not exactly the same, we have worked with quite a bit. Setting intermediate goals, as was done, was exciting. However, there are some aspects we are not 100% satisfied with, including the inclusion of the school in the goal setting for the ‘step-ladder’, for example, which steps to take.” (P7).

Moreover, several informants highlighted that the B2S team were external partners who could help parents at home, which was different from “practice as usual,” such as helping parents with sleeping habits and interventions for the mothers. The findings suggest that this work also helped achieve a more common understanding among all parties involved.

The involvement and prioritization of the work by department heads was recognized by several informants:

“But we do have the Head of the Department in the teams (grade-level) meetings. I brought up this student in several grade-level meetings, so they were informed all the way about what we offer and what we think we need to try, and they supported the measures we tried.” (P4).

Another informant added:

“We often discussed with the principal. He was frequently involved in conversations about the student. I believe the principal has attended all the meetings we have had previously regarding the absenteeism issue.” (P6).

All the informants experienced that the B2S team was receptive to and included the school perspective, and they were good discussion partners. They also experienced that the B2S team acknowledged what the school had done before the intervention. Informants found the B2S team to be excellent in communication with parents and the school, respecting the school’s framework, contributions, and roles. The experience of including the school’s perspectives was perceived as new in B2S compared to interactions with external services before B2S, as illustrated by Informant 4:

“Yes, I think it provides more perspectives around the parents. I also believe the parents need a channel to talk about the absenteeism and what is happening at school with professionals, which I think is very useful.” (P4).

Several informants expressed that the school’s perspectives and experiences were included in the “common understanding.” However, the parents and B2S team could come to the school meetings with a plan, but the school members often felt unprepared and merely received information—they reported that they should have been prepared on themes and interventions. Two informants felt they were dictated to by B2S representatives and wished they were more involved throughout the project:

“We felt that we were not part of setting them… The goals were given to us, not something we were involved in.” (P7).

In some cases, the school and parents had different views about reasons and problems before starting in B2S, such as how much should be required and adapted for. For example, the process of information gathering, analysis, and assessment to understand the student’s situation made the attendance problems clearer. The informants emphasized the importance of a common understanding and cooperation between the involved parties. It was good for the teachers to know what interventions were occurring at home and that the parents felt safe about the school’s intervention. However, some felt that the school did too much or “stretched too far”:

“What we tried was having several conversations with the parents, holding several meetings with them. And directly towards the student, we tried to adapt the teaching. The student has missed quite a bit of instruction and had more absenteeism than… We adapted the teaching a bit, we had the opportunity to use group rooms for the student to withdraw a little. And then it kind of stalled before the B2S project. We probably had different views on it, we and the parents, regarding how much we should push. This led to poor communication. They felt that we did not listen to the supervisor, and we felt that they should have pushed more. I think the common understanding somewhat cleared up things when we started this project.” (P6).

Some informants considered it a strength having members in the B2S team, who worked in Educational and Psychological Services (EPS), because the relationships were in place before the intervention, making the student feel safer. While cooperation and communication with the B2S team was experienced as good, some informants felt that the school sessions should have included more school personnel:

“There has been very close follow-up. The home-school collaboration has been closely monitored since the start of 4th grade level. EPS was involved in relation to absenteeism issues already then, and they have been a close partner. It has been a bit up and down, with periods that have been good, but the case is not closed. It was open all the way until the student started B2S this fall. It is the same contact person we had in EPS, who is a part of the B2S intervention today. I would say that has definitely been a strength. Since B2S is so compressed in terms of time span, the relationship was established in advance. The student was also more comfortable in the conversations. I would definitely say that has been a strength and an advantage for the student.” (P7).

#### Workload

The informants experienced a significant workload for the schools related to planning and implementing interventions, especially as none of them had extra resources for this work. All informants said it was difficult to differentiate between the school sessions and school meetings and could not provide specific information about these meetings and sessions separately. They had varied experiences with the number of meetings, from too many to too few, but most were pleased with the number and content, even though they felt the content was often a repetition of what they already knew and did before.

“I felt that it increased our competence. There were things we had heard before, but it was very good to have them repeated. It was very good that all of us from school, together, got to hear it, that we had a common language. And again, we received recognition that we had done this and that we had experience with it, it was a very good reminder.” (P3).

As expected, nearly all informants pinpointed the dilemmas of giving so much attention to one pupil when the class consisted of up to 25 students.

“There was a very one-sided focus on this student without considering us as contact teachers in a class to relate to. It should be said that we have used an incredible number of resources on the student without additional substitute teachers or other resources being provided. We have had to take special education hours from other students, and that is not fortunate. I will not say it is bad. But it is not good.” (P7).

Another informant illustrated a similar dilemma:

“Yes. It is very challenging to schedule so many meetings. I have 25 students in a group, and when you have 4–5 students with attendance problems, it takes a lot of time to prepare for subjects and assessments.” (P4).

#### School sessions and school meetings

School sessions were meetings without parents, while school meetings included parents. Five of the seven informants experienced differences between school sessions and school meetings, noting they could speak more freely about school experiences without the parents present. One informant felt the meetings with parents were necessary because of a specific parent dynamic but also noted that both parents attended together with the child (suggesting separation anxiety was still being addressed). Conversely, another informant felt open discussion was possible with the parents present in the meetings. Clearly, the informants experienced this differently. Two illustrations from the informants highlight this.

“I find that it has been very good support. It has been very beneficial to have someone outside the school who is working with school absenteeism. We often sit at school with the best offerings/interventions in the world, but the students do not come to us. So having someone who has been in the home working with sleep, for example, and being able to collaborate and support the mother has been invaluable. Plus, it has been very good to receive recognition that we are doing a good job and providing examples of things we can try. It was very good. It is good not to be alone.” (P3).

Further from another informant:

“It becomes easier to discuss the most difficult things and hear a third party’s view on it before we have the discussion together. I think it is more organized, and I think it is better for the collaboration with the parents as well. It probably applies to their part too.” (P6).

### Impact of B2S participation

#### Teachers’ competence and self-efficacy

Most informants expressed that the content of the B2S intervention for the school was mostly a confirmation or acknowledgment of what was known before. However, some increased their competence during the intervention. One informant explained the work with school attendance problems and experienced that no cases are the same and that there are no measures that fit all:

“Even though I have experiences with this from many years earlier, I find that all cases are so unique and different, so I feel it is difficult to use previous experience on new cases. And that they are usually very complex cases. I do not feel that it is something I feel confident about, no. It’s not something where I think I have the solution or know how we should work here. It’s a bit of trial and error and groping in the dark with these types of cases. But then I find that even though we have several parallel cases that also have some similarities, the measures will be so different and have different effects. And communication is so different that it is difficult to see what you can take out or carry forward. And if I am to say something about mastery, this student has not, despite all the measures, despite such a good setup/adaptation around the student, improved school attendance.” (P4).

Concrete examples presented as useful by the informants included the use of a “step-ladder,” reward systems, clear goals, and the collaboration method. The informants said that their self-efficacy regarding their current case was primarily based on the results for the participating student; if the student attended school more, their self-efficacy was better. Several informants claimed that no cases were similar, and what helps one student does not help others, as highlighted in the previous quote from P4.

#### Students’ attendance

An important outcome of this intervention was to increase students’ school attendance. Based on the answers of the seven informants, three of the students attended school more after the B2S intervention, two did not attend school more, one student attended school but only with parents in immediate presence, and one student transferred at a new school.

However, the informants claimed that there were other impacts of the intervention as well. Some of these impacts included that the student and parents learned more about mastery skills and different tools for this, and that the parents received help and other perspectives to understand and handle the situation. In the long run, this support can help the informants cope with the situation, and external partners like the B2S team provided emotional support. Moreover, some informants expressed a need for a longer intervention period than 3 months, and some students were referred to other services after the intervention. Here are some illustrations underpinning this theme:

“The parents showed up, and we never got to test the measures (interventions). It became more about how the student talks more with the parents now than before, talking with the B2S team, yes. So, it became a bit indirect. An indirect communication with the student. They told us what the student had said to them.” (P1).

“I think it comes from the student receiving help to handle other challenges, which has made some pieces fall into place… For example, getting further referrals and that has yielded results that make some pieces fall into place. I also know that the student has greatly benefited from the ‘what can happen’ mindset.” (P2).

Informants P3 and P6 reported higher attendance:

“Yes, I do feel that the student attends school more now, but Jan is not completely back. You get a good feeling when Jan attends school more. But there is still quite a bit of absenteeism.” (P3).

“Yes. I feel Janne has come a long way now. Janne is basically back to how we knew them before.” (P6).

### Feedback on B2S

#### Success factors

When asked what they experienced as important in the B2S intervention, several informants mentioned that it was beneficial to know one of the people in the B2S team before the intervention, as some members were personnel from Educational and Psychological Services (EPS).

“There has been very close follow-up. The home-school collaboration has been closely monitored since the start of 4th grade level. EPS was involved in relation to absenteeism issues already then, and they have been a close partner. It has been a bit up and down, with periods that have been good, but the case is not closed. It has been open all the way until they started in B2S this fall. It is the same contact person we had in EPS who is a part of the B2S team now.” (P7).

Two informants mentioned the importance of teachers’ motivation and involvement from the start (during the recruitment period), as the teacher is the main contributor in implementing the interventions/measures at school. One of them emphasized:

“We think that a prerequisite for something to work at school is that we at the school get to choose who it is appropriate to collaborate with, and then I am thinking about having a contact teacher who is motivated. The contact teacher here was very clear that this was not something she was motivated to participate in, she said that clearly in the meeting with B2S… So, if she had been involved in the process and chosen who could be a suitable candidate, we would have chosen another student.” (P5).

Three informants talked about the importance of providing information about the B2S intervention in school team meetings for all school personnel, to achieve a more holistic school approach and a more robust intervention. However, this was reportedly missing according to several informants. When asked how their fellow colleagues at school were involved in bringing information about the participating student’s situation, one answered:

“To a small extent, because I am the main teacher, I am the one who has most of the information. But if I approach other teachers in specific subjects, I do get it.” (P7).

#### Barriers

The informants’ experiences varied significantly when asked about what was missing or the barriers they experienced during the intervention. Time and workload to participate in the meetings/sessions and to fill out questionnaires were mentioned by most of them. Moreover, they did not have extra resources to participate in the intervention. One informant said:

“School absenteeism should give more resources.” (P7).

This informant (P7) argued that the intervention required a lot of extra work to help the student with absenteeism problems. One informant mentioned that the school did too much (“stretched too far”), which might have affected the rest of the students in class. Two informants felt they were not involved when the B2S team and parents planned the intervention, even though the school had to make adaptations for the student. Another informant said they lost control and did not have “the hand on the wheel” (P5) until the case was excluded due to a reported unsafe learning environment for the student (in line with the Education Act §12–2). In that situation, the school had to make a plan to ensure the student’s safe learning environment. One informant experienced that the included student was too complex a case, and even the B2S intervention was not helpful for the student:

“This was a very difficult case, even the B2S team could not help the student back to school even though they had competence.” (P1).

Another informant felt impatient as they wanted to move faster for the student to attend school. One informant mentioned too much focus on the student’s voice, and that this student decided too much. Two of them talked about their need to have more information about the B2S-manual and the ongoing work to be better prepared for the school meetings. One mentioned the importance of involving the school’s leadership and the need for more group rooms in school. Another informant expressed a need for the B2S team to observe the students and give feedback on success and risk factors in the classroom. One informant wanted more competence regarding mental health problems like anxiety, illustrating the difficulties and dilemmas:

“I would assume that there are a lot of psychological aspects involved and more of the psychological part so that one can understand more of the background for it. That would probably help… I am an educator, I am neither a doctor, nurse, nor psychologist, so that part is quite difficult for a teacher to know how to solve without getting help. The support services are under pressure and the management is often under pressure. There is something about actually feeling that you get a bit of help to handle it.” (P1).

## Discussion

The main findings from this study indicated that some informants reported higher competence and self-efficacy in working with absenteeism following the intervention, while other teachers did not have the same experience. The informants experienced a limited overview regarding the stages of the intervention, and reported they needed more clarity regarding the role and content of the school before, during, and after the intervention. However, the competence and support from the B2S team was highly appreciated among the informants.

To answer the research question for this study, “How do teachers experience their participation in the Back2School intervention?,” we start discussing the results concerning the informants’ experience of higher competence and self-efficacy after participating in the B2S pilot intervention. The informants’ self-efficacy was primarily based on their particular student’s attendance rates, which is in line with previous research (Gale et al., 2021). When their student did not increase school attendance rates, this influenced the informants’ self-efficacy, where they felt that their efforts did not help (Gale et al., 2021). Conversely, when the student attended school more, their self-efficacy increased, as they found working with students having school absenteeism problems complex and challenging (Cunningham et al., 2022). Only three of the seven informants reported higher students’ school attendance after the intervention, which could be related to the need for a longer intervention period than 3 months, and that some of the students had too complex problems, requiring referral to other services after the intervention period. Duration of the intervention is one of the important factors found in the review by Boaler and Bond (2023). It is also important to bear in mind that the informants expressed the intervention might have impacted other outcomes, where the student and parents learned about mastery skills, different tools for dealing with difficulties, and parents’ experience of receiving help and new perspectives, as suggested by Cunningham et al. (2022). This outcome might help the student and parents to cope better with the situation, and a qualified B2S team can help them discuss their situation and support their feelings when having a child at home struggling with attending school (Kearney and Graczyk, 2022).

Teachers’ competence and self-efficacy influence the success of all interventions in school, like B2S. When teachers experience confidence in their abilities to support students, they are more likely to engage actively in B2S activities. Professional development opportunities can enhance teachers’ skills and boost their confidence, leading to more effective implementation of B2S. Several informants stated that they received confirmation and acknowledgment of the work they did before the pilot by the B2S team, as well as their prior competence. As the informants expressed that participation in the pilot intervention mostly confirmed their existing knowledge, one might question why the students developed problematic absenteeism. This could be explained by all the possible risk factors for absenteeism, the complexity of the problems (Gubbels et al., 2019; Lomholt et al., 2022), and the need for a holistic approach to understand and intervene (Heyne and Brouwer-Borghuis, 2022). Moreover, if schools are equipped to work in a holistic way such as the B2S intervention is based on (family, student, and school), there can be better opportunities to reduce absenteeism (Kearney and Graczyk, 2022).

Although the findings indicate mixed results in regard to the informants’ experiences of learning something new during the intervention, some of the informants expressed that they learned some new, useful, and concrete “tools.” These were related to CBT and were not typical tools for the school setting or for teachers, as this is not part of their education or mandate. Despite this, all teachers in Norway should incorporate some of these CBT skills in all lessons, since it is part of the core curriculum in the Education Act, including a section on public health and life skills. This emphasizes the importance of equipping students with knowledge and skills necessary to promote good physical and mental health, and to make responsible life choices (Avdem, 2025; UDir, 2025).

An important factor in the results was the lack of an overview of the stages in the B2S intervention among the informants. The informants stated the importance of teachers’ motivation and involvement from the start (recruitment), as teachers are the main contributors in implementing the interventions at school. All informants reported a lack of understanding differentiating the school meetings and school sessions. Despite the benefits of participating in B2S, several barriers can hinder effectiveness. This may include limited resources and a lack of time for planning. Identifying and addressing these barriers is essential for maximizing the impact of B2S. The informants mentioned some of the barriers to be a lack of time and too much workload, both for completing questionnaires and participating in the school meetings and sessions. This dilemma is also emphasized in previous research (Cunningham et al., 2022; Finning et al., 2018), where time spent on one student was emotionally demanding and time-consuming and at the expense of the whole class. Bearing in mind the significant potential risk factors (Gubbels et al., 2019; Lomholt et al., 2022), where school domains such as student-teacher relationship, low quality of school/education, and a negative school/class climate had a large effect on school absenteeism, which underpins the importance of sufficient resources. Kearney et al. (2023) argues that school absenteeism is influenced by student, parent, family, peers, school, and community factors, and is caused by multiple factors where the key influential factors are interrelated.

Three of the informants expressed the importance of giving information about the B2S intervention to the rest of the school staff, as shared information provided an opportunity for a more holistic school approach and robust intervention. However, the latter was reportedly missing according to several informants. The importance of school involvement and reducing the barriers for schools to be an integrated part of the intervention is related to the importance of implementing CBT in the school environment and incorporating a school-based component (Fernandes et al., 2024). Based on their review, they recommended further exploration and development of interventions with an integral school-based component, which was done in the B2S pilot, but the findings from the current study indicate a need for better integration and a larger school-based component to better help students with absenteeism problems.

The lack of overview during the intervention is closely related to a lack of clarity regarding the school role and content. The informants experienced the school’s role and involvement with a lack of clarity regarding their participating role in the intervention. This is in line with Hejl et al. (2024), where the lack of resources and competencies within the school system, unclear roles and division of responsibilities, and lacking collaboration act as obstacles to effective management. Teachers emphasize the need for joint, interdisciplinary collaboration with external professionals and call for more structured support and guidance, and they believe that involving a third-party mediator could improve absenteeism management (Hejl et al., 2024). In the B2S pilot, the third party was the B2S team, which many of the informants appreciated.

Collaboration and involvement from a group of external professionals (B2S team) were valued among the informants, as the members of the B2S team had competence and experience with absenteeism problems, were good discussion partners, had good communication with both parents and the school, respected the school’s framework and the school’s contributions and roles, and provided acknowledgment for what they did for the student prior to the pilot study. Previous research indicates that school personnel find this topic complex and challenging (Cunningham et al., 2022), and it is understandable that they appreciate getting help from externally qualified professionals. Moreover, interdisciplinary collaboration with external professionals has been previously suggested in research (Hejl et al., 2024; Nielsen and Thastum, 2023).

Even though the informants appreciated being part of the pilot, they spent a lot of time participating, as they did not have extra resources to take part in the pilot intervention. Most of the informants expressed that they did not do anything different during the pilot than before. However, several informants appreciated the help in supporting the student, and the work tool “step-ladder” was most frequently mentioned as helpful. Additionally, several informants expressed that the school’s perspectives and experiences were included in the “common understanding,” which is an important part of the intervention. Findings from a systematic review (Fernandes et al., 2024) show that interventions need to involve school-based components to succeed. The informants in the current study were not always prepared for the school meetings, as the B2S team and parents had sometimes made plans for school interventions in advance without the school’s involvement, leaving some with a sense of being told what to do and losing control. These informants did not feel included in the whole process. School involvement is important from both parents’ and teachers’ views (Lomholt et al., 2020; Nielsen and Thastum, 2023), but several informants in the current study felt left out.

### Strengths and limitations

The sufficiency of data from a sample of 7 informants depends on the study’s aim, interview quality, and the analysis strategy chosen (Bouncken et al., 2025; Malterud et al., 2016). In the current study, while the quality of the interview dialogues varied somewhat depending on how comprehensive the answers provided by the informants were, we achieved a good breadth regarding the informants’ professional and experience backgrounds, which provided more perspectives on the issues. Nevertheless, only half of the teachers who took part in the B2S intervention agreed to participate in the current interview study. Therefore, we are not sure whether the non-participating teachers would give other relevant insight to answer the research question. This limitation is of importance for this study.

Both interviewers have been working with school absenteeism over years, and both were actively involved with meta supervision of the B2S teams. The interviewers interviewed teachers who were from another district than those they supervised, to avoid bias by having extensive knowledge about the cases the teachers referred to. During the intervention the interviewers had no direct contact with the informants they interviewed in this study.

Another limitation which is important, is that we did no member check (participant validation) which could ensure the accuracy and credibility of the data.

Furthermore, the study was conducted in Norway, and the context might not be transferable to other countries, as the Nordic countries have an Education Model comprising a compulsory school system and emphasizing “A School for All” (Blossing et al., 2014; Juusola, 2023). This model emphasizes equal opportunities provided for all students regardless of cultural, social, and economic background (Juusola, 2023; Klette et al., 2018). There is evidence that countries with similar cultures and educational policies have similar educational results (Nilsen and Gustafsson, 2016). This is important to keep in mind when comparing results between different school contexts and countries within and outside the Nordic countries. Although Denmark (where the B2S intervention is developed and evaluated) is also part of the Educational Model, there are differences in these school systems as well. In Norway, education as such is obligatory, while in Denmark attending school is obligatory. Moreover, in Denmark more students attend special schools than in Norway (8,359 vs. 6,000 students in the school year 2020/21) (EASIE, 2025).

### Future research directions

Building on the evidence from the current and previous studies, there is a clear need to further develop intervention content that enhances school and teachers’ participation and facilitates better adaptations for students in school. Future research should prioritize the integration of individual and systemic components within school attendance interventions. At present, these components often operate in parallel, lacking the cohesion that many informants have identified as essential for improving intervention outcomes. The upcoming RCT of the B2S intervention in Norway will take this into account, aiming to test a more integrated and holistic approach to support school attendance.

## Conclusion

The informants, six regular teachers and one from the Head of Department, who participated in the pilot Back2School Norway, highlighted the need for a clear structure and a predictable process for all parties involved. The informants felt they had the competence and experience to identify students suitable for the intervention. Moreover, they stated a need for better clarity regarding their participation throughout the entire intervention. The informants wanted to be thoroughly involved and engaged in the whole intervention process. Some of the informants experienced higher self-efficacy and competence in working with students experiencing school absenteeism, while others did not. All informants emphasized the crucial importance of close cooperation with the student, parents, and external support from the B2S team. They particularly valued the B2S team’s knowledge and experience, which could help the teachers, the student, and the parents. However, challenges related to workload, resource allocation, and the degree of genuine involvement need attention for future implementations.

## Data Availability

The datasets presented in this article are not readily available because restrictions apply to the availability of the data due to its sensitive nature and ethical restrictions. Data are available from the authors upon reasonable request. Requests to access the datasets should be directed to elisabeth.bania@ntnu.no.

## References

[ref1] Akkus M. Çinkir S. (2022). The problem of student absenteeism, its impact on educational environments, and the evaluation of current policies. Int. J. Psychol. Educ. Stud. 9, 978–997. doi: 10.52380/ijpes.2022.9.4.957

[ref2] Archambault I. Janosz M. Olivier E. Dupéré V. (2022). “Student engagement and school dropout: theories, evidence, and future directions” in Handbook of research on student engagement. eds. Reschly A. Christenson S.. (Switzerland AG: Springer), 331–355. doi: 10.1007/978-3-031-07853-8_16

[ref3] Avdem J. (2025). Folkehelse og livsmestring i skolen–kompetanse eller danning? [Public Health and Life Skills in School – Competence or Bildung?]. Nord. Tidsskr. Pedagog. Krit. 11, 256–271. doi: 10.23865/ntpk.v11.6840

[ref4] Balfanz R. Byrnes V. (2012). The importance of being there: a report on absenteeism in the nation’s public schools (Baltimore, MD: Johns Hopkins University School of Education, Everyone Graduates Center, Get Schooled, Issue. Available online at: https://www.attendanceworks.org/wp-content/uploads/2017/06/FINALChronicAbsenteeismReport_May16.pdf

[ref5] Bernacki M. L. Greene M. J. Lobczowski N. G. (2021). A systematic review of research on personalized learning: personalized by whom, to what, how, and for what purpose (s)? Educ. Psychol. Rev. 33, 1675–1715. doi: 10.1007/s10648-021-09615-8

[ref6] Blossing U. Imsen G. Moos L. (2014). The Nordic education model. Dordrecht: Springer Netherlands.

[ref7] Boaler R. Bond C. (2023). Systemic school-based approaches for supporting students with attendance difficulties: a systematic literature review. Educ. Psychol. Pract. 39, 439–456. doi: 10.1080/02667363.2023.2233084

[ref8] Bouncken R. B. Czakon W. Schmitt F. (2025). Purposeful sampling and saturation in qualitative research methodologies: recommendations and review. Rev. Manag. Sci. 1–37. doi: 10.1007/s11846-025-00881-2

[ref9] Braun V. Clarke V. (2006). Using thematic analysis in psychology. Qual. Res. Psychol. 3, 77–101. doi: 10.1191/1478088706qp063oa

[ref10] Chang H. N. Bauer L. Byrnes V. (2018). Data matters: using chronic absence to accelerate action for student success. Executive summary. Attendance works, issue. Available online at: www.attendanceworks.org/

[ref11] Chu B. C. Guarino D. Mele C. O’Connell J. Coto P. (2019). Developing an online early detection system for school attendance problems: results from a research-community partnership. Cogn. Behav. Pract. 26, 35–45. doi: 10.1016/j.cbpra.2018.09.001

[ref12] Clarke V. Braun V. (2021). Thematic analysis: a practical guide. Torrossa Digital Online Bookstore. London: SAGE Publications Ltd.

[ref13] Cohen L. Manion L. Morrison K. (2002). Research methods in education. London: Routledge. doi: 10.4324/9780203224342

[ref14] Cunningham A. Harvey K. Waite P. (2022). School staffs’ experiences of supporting children with school attendance difficulties in primary school: a qualitative study. Emot. Behav. Diffic. 27, 72–87. doi: 10.1080/13632752.2022.2067704

[ref15] Department for Education. (2024). Pupil absence in schools in England, Academic year 2022/23 – Explore education statistics. Available online at: https://explore-education-statistics.service.gov.uk/find-statistics/pupil-absence-in-schools-in-england

[ref16] EASIE. (2025). Available online at: https://www.european-agency.org/activities/data/cross-country-reports

[ref17] Elliott J. G. Place M. (2019). Practitioner review: school refusal: developments in conceptualisation and treatment since 2000. J. Child Psychol. Psychiatry 60, 4–15. doi: 10.1111/jcpp.12848, PMID: 29197106

[ref18] Fernandes C.-S. F. Kannoth S. Pendergrass Boomer T. M. Hieftje K. D. Fiellin L. E. (2024). Systematic review of interventions with some school involvement for school refusal in high school–age adolescents. Child. Sch. 46:cdae003. doi: 10.1093/cs/cdae003

[ref19] Finning K. Harvey K. Moore D. Ford T. Davis B. Waite P. (2018). Secondary school educational practitioners’ experiences of school attendance problems and interventions to address them: a qualitative study. Emot. Behav. Diffic. 23, 213–225. doi: 10.1080/13632752.2017.1414442 PMC7254525 32536954

[ref20] Fredriksson U. Rasmusson M. Backlund Å. Isaksson J. Kreitz-Sandberg S. (2023). School absenteeism among students in Germany, Japan, Sweden, and the United Kingdom: a comparative study using PISA data. Nordic J. Comp. Int. Educ. 7, 1–27. doi: 10.7577/njcie.5034

[ref21] Gale J. Alemdar M. Cappelli C. Morris D. (2021). A mixed methods study of self-efficacy, the sources of self-efficacy, and teaching experience. Front. Educ. 6:750599. doi: 10.3389/feduc.2021.750599

[ref22] Gottfried M. Little M. Ansari A. (2024). Novice teachers and student attendance in early elementary school. Educ. Policy 39:08959048241231952. doi: 10.1177/08959048241231952

[ref23] Graczyk P. A. Kearney C. A. (2024). Roadmap for implementing a multi-tiered system of supports framework to improve school attendance. Curr. Psychol. 43, 15286–15307. doi: 10.1007/s12144-023-05478-0

[ref24] Gren-Landell M. Ekerfelt Allvin C. Bradley M. Andersson M. Andersson G. (2015). Teachers’ views on risk factors for problematic school absenteeism in Swedish primary school students. Educ. Psychol. Pract. 31, 412–423. doi: 10.1080/02667363.2015.1086726

[ref25] Gubbels J. van der Put C. E. Assink M. (2019). Risk factors for school absenteeism and dropout: a meta-analytic review. J. Youth Adolesc. 48, 1637–1667. doi: 10.1007/s10964-019-01072-5, PMID: 31312979 PMC6732159

[ref26] Havik T. Bru E. Ertesvåg S. K. (2014). Parental perspectives of the role of school factors in school refusal. Emot. Behav. Diffic. 19, 131–153. doi: 10.1080/13632752.2013.816199

[ref27] Havik T. Ingul J. M. (2021). How to understand school refusal. Front. Educ. 6:715177. doi: 10.3389/feduc.2021.715177

[ref28] Hejl C. Ezzaaf Fryland N. Hansen R. B. Nielsen K. Thastum M. (2024). A review and qualitative synthesis of the voices of children, parents, and school staff with regards to school attendance problems in the Nordic countries. Scand. J. Educ. Res. 68, 1–15. doi: 10.1080/00313831.2024.2434822, PMID: 40640861

[ref29] Heyne D. (2022). Practitioner review: signposts for enhancing cognitive-behavioral therapy for school refusal in adolescence. Z. für Kinder Jugendpsychiatrie Psychother. 51, 61–76. doi: 10.1024/1422-4917/a000899 36111580

[ref30] Heyne D. Brouwer-Borghuis M. (2022). Signposts for school refusal interventions, based on the views of stakeholders. Contin. Educ. 3, 25–40. doi: 10.5334/cie.42, PMID: 38774290 PMC11104337

[ref31] Heyne D. A. Sauter F. M. Maynard B. R. (2015). Moderators and mediators of treatments for youth with school refusal or truancy. EBSCO Publishing: ebook Collection (EBSCO host). 230–266. doi: 10.1093/med:psych/9780199360345.003.0010

[ref32] Hunter D. McCallum J. Howes D. (2019). Defining exploratory-descriptive qualitative (EDQ) research and considering its application to healthcare. J. Nurs. Health Care 4.

[ref33] Johnsen D. B. Lomholt J. J. Heyne D. Jensen M. B. Jeppesen P. Silverman W. K.. (2024). The effectiveness of modular Transdiagnostic cognitive behavioral therapy versus treatment as usual for youths displaying school attendance problems: a randomized controlled trial. Res. Child Adolesc. Psychopathol. 52, 1397–1412. doi: 10.1007/s10802-024-01196-8, PMID: 38739306 PMC11420258

[ref34] Josefowitz N. Myran D. (2021). CBT made simple: a clinician's guide to practicing cognitive behavioral therapy. Oakland, CA: New Harbinger Publications.

[ref35] Juusola H. (2023). What do young people think about the extension of compulsory education? Educ. Res. 65, 478–495. doi: 10.1080/00131881.2023.2263885

[ref36] Kearney C. (2021). Integrating systemic and analytic approaches to school attendance problems: synergistic frameworks for research and policy directions. Child Youth Care Forum 50, 701–742. doi: 10.1007/s10566-020-09591-0

[ref37] Kearney C. A. Benoit L. Gonzálvez C. Keppens G. (2022). School attendance and school absenteeism: a primer for the past, present, and theory of change for the future. Front. Educ. 7, 1–17. doi: 10.3389/feduc.2022.1044608, PMID: 40709351

[ref38] Kearney C. A. Childs J. (2023). Improving school attendance data and defining problematic and chronic school absenteeism: the next stage for educational policies and health-based practices. Prev. Sch. Fail. Altern. Educ. Child. Youth 67, 265–275. doi: 10.1080/1045988X.2022.2124222

[ref39] Kearney C. A. Dupont R. Fensken M. Gonzálvez C. (2023). School attendance problems and absenteeism as early warning signals: review and implications for health-based protocols and school-based practices. Front. Educ. 8, 1–15. doi: 10.3389/feduc.2023.1253595

[ref40] Kearney C. A. Graczyk P. A. (2020). A multidimensional, multi-tiered system of supports model to promote school attendance and address school absenteeism. Clin. Child. Fam. Psychol. Rev. 23, 316–337. doi: 10.1007/s10567-020-00317-1, PMID: 32274598

[ref41] Kearney C. A. Graczyk P. A. (2022). Multi-tiered systems of support for school attendance and its problems: an unlearning perspective for areas of high chronic absenteeism. Front. Educ. 7, 1–5. doi: 10.3389/feduc.2022.1020150, PMID: 40709351

[ref42] Kirksey J. J. (2019). Academic harms of missing high school and the accuracy of current policy thresholds: analysis of preregistered administrative data from a California school district. AERA Open 5:2332858419867692. doi: 10.1177/2332858419867692

[ref43] Klette K. Sahlström F. Blikstad-Balas M. Luoto J. Tanner M. Tengberg M.. (2018). Justice through participation: student engagement in Nordic classrooms. Educ. Inq. 9, 57–77. doi: 10.1080/20004508.2018.1428036

[ref44] Lomholt J. J. Arendt J. N. Bolvig I. Thastum M. (2022). Children with school absenteeism: comparing risk factors individually and in domains. Scand. J. Educ. Res. 66, 411–426. doi: 10.1080/00313831.2020.1869085

[ref45] Lomholt J. J. Johnsen D. B. Silverman W. K. Heyne D. Jeppesen P. Thastum M. (2020). Feasibility study of Back2School, a modular cognitive behavioral intervention for youth with school attendance problems. Front. Psychol. 11:491629. doi: 10.3389/fpsyg.2020.00586 PMC7153503 32328012

[ref46] Malcolm H. Wilson V. Davidson J. Kirk S. (2003). Absence from school: a study of its causes and effects in seven LEAs. The SCRE Centre University of Glasgow. Glasgow.

[ref47] Malterud K. (2002). Qualitative methods in medical research-preconditions, potentials and limitations. Tidsskr. Nor. Laegeforen. 122, 2468–2472. 12448119

[ref48] Malterud K. Siersma V. D. Guassora A. D. (2016). Sample size in qualitative interview studies: guided by information power. Qual. Health Res. 26, 1753–1760. doi: 10.1177/1049732315617444, PMID: 26613970

[ref49] Melvin G. McKay-Brown L. Heyne D. Cameron L. (2025) Barriers to school attendance and reasons for student absence: Rapid literature review (192306648X). Available online at: https://www.edresearch.edu.au/research/research-reports/barriers-school-attendance-and-reasons-student-absence

[ref50] Nazar Z. J. Nazar H. Rainkie D. El-Awaisi A. ElJaam M. (2023). “Evidence produced while using qualitative methodologies including research trustworthiness” in Encyclopedia of evidence in pharmaceutical public health and health services research in pharmacy. (Switzerland AG: Springer), 699–712. doi: 10.1007/978-3-030-64477-2_76

[ref51] Nielsen K. Thastum M. (2023). School absence seen from a school perspective and a parent perspective. Nordic Stud. Educ. 43, 325–343. doi: 10.23865/nse.v43.4080

[ref001] Nilsen T. Gustafsson J. E. (2016). Teacher quality, instructional quality and student outcomes: Relationships across countries, cohorts and time. Springer International Publishing AG Switzerland. doi: 10.1007/978-3-319-41252-8

[ref52] Pérez-Marco M. Gonzálvez C. Fuster-Rico A. Vicent M. (2024). A systematic review of intervention programs for school attendance problems. Child Youth Serv. Rev. 169:108091. doi: 10.1016/j.childyouth.2024.108091, PMID: 40704057

[ref53] Stalmeijer R. E. Brown M. E. O'Brien B. C. (2024). How to discuss transferability of qualitative research in health professions education. Clin. Teach. 21:e13762. doi: 10.1111/tct.13762, PMID: 38497107

[ref54] Thastum M. Arendt K. B. Kjerholt C. M. (2020). Back2School: Manual til behandling af børn med bekymrende skolefravær (manual for treatment of youths with problematic school absenteeism). CEBU - Center for Psykologisk Behandling til Børn og Unge. Psykologisk Institut. Aarhus Universitet, DK. Available at: https://psy.au.dk/en/cebu

[ref55] Thastum M. Johnsen D. B. Silverman W. K. Jeppesen P. Heyne D. A. Lomholt J. J. (2019). The Back2School modular cognitive behavioral intervention for youths with problematic school absenteeism: study protocol for a randomized controlled trial. Trials 20, 29–12. doi: 10.1186/s13063-018-3124-3, PMID: 30621787 PMC6325742

[ref56] Thompson J. (2022). A guide to abductive thematic analysis. Qual. Rep. 27, 1410–1421. doi: 10.46743/2160-3715/2022.5340, PMID: 39386316

[ref57] UDir. (2023). Statistics school absence 10th grade Norway, 2022–2023. Available online at: https://www.udir.no/tall-og-forskning/statistikk/statistikk-grunnskole/analyser/2023/fravar-pa-10.-trinn-skolearet-22-23/#:~:text=Elevene%20p%C3%A5%2010.%20trinn%20har%20typisk%20%C3%A5tte%20dager,dag%20og%20to%20enkelttimer%20h%C3%B8yere%20frav%C3%A6r%20enn%20guttene

[ref58] UDir. (2025 Core curriculum – values and principles for primary and secondary education). Available online at: https://www.udir.no/lk20/overordnet-del/?lang=eng

[ref59] Weeks C. Hill V. Owen C. (2017). Changing thoughts, changing practice: examining the delivery of a group CBT-based intervention in a school setting. Educ. Psychol. Pract. 33, 1–15. doi: 10.1080/02667363.2016.1217400

[ref60] Wilson V. Malcolm H. Edward S. Davidson J. (2008). ‘Bunking off’: the impact of truancy on pupils and teachers. Br. Educ. Res. J. 34, 1–17. doi: 10.1080/01411920701492191

